# Change in condom and other barrier method use during and after an HIV prevention trial in Zimbabwe

**DOI:** 10.1186/1758-2652-13-39

**Published:** 2010-10-19

**Authors:** Ariane van der Straten, Helen Cheng, Alexandra M Minnis

**Affiliations:** 1 Women's Global Health Imperative, RTI international, San Francisco Project Office, San Francisco, CA, USA; 2 University of California San Francisco, Center for AIDS Prevention Studies, Department of Medicine, San Francisco, CA, USA; 3 University of California Berkeley, School of Public Health, Berkeley, CA, USA

## Abstract

**Background:**

We examined the use of male condoms and the diaphragm following completion of a clinical trial of the diaphragm's HIV prevention effectiveness. In the trial, called Methods for Improving Reproductive Health in Africa (MIRA), women were randomized to a diaphragm group (diaphragm, gel and condoms) or a condom-only control group. At trial exit, all women were offered the diaphragm and condoms.

**Methods:**

Our sample consisted of 801 Zimbabwean MIRA participants who completed one post-trial visit (median lapse: nine months; range two to 20 months). We assessed condom, diaphragm and any barrier method use at last sex act at enrolment, final MIRA and post-trial visits. We used multivariable random effects logistic regression to examine changes in method use between these three time points.

**Results and discussion:**

In the condom group, condom use decreased from 86% at the final trial visit to 67% post trial (AOR = 0.20; 95% CI: 0.12 to 0.33). In the diaphragm group, condom use was 61% at the final trial visit, and did not decrease significantly post trial (AOR = 0.77; 95% CI: 0.55 to 1.09), while diaphragm use decreased from 79% to 50% post trial (AOR = 0.18; 95% CI: 0.12 to 0.28). Condom use significantly decreased between the enrolment and post-trial visits in both groups. Use of any barrier method was similar in both groups: it significantly decreased between the final trial and the post-trial visits, but did not change between enrolment and the post-trial visits.

**Conclusions:**

High condom use levels achieved during the trial were not sustained post trial in the condom group. Post-trial diaphragm use remained relatively high in the diaphragm group (given its unknown effectiveness), but was very low in the condom group. Introducing "new" methods for HIV prevention may require time and user skills before they get adopted. Our findings underscore the potential benefit of providing a mix of methods to women as it may encourage more protected acts.

## Background

Condom promotion is a central component of a comprehensive prevention package offered to participants in HIV prevention trials of female-initiated barrier methods. Long-term effects of such intensive promotion on sustained condom use after trial completion is a critical yet insufficiently examined area as it could inform condom rollout programmes, as well as operations research after demonstration of new, successful biomedical interventions. To our knowledge, only one study examined condom use prevalence following participation in a sexually transmitted infection (STI) and HIV prevention trial. This trial of nonoxynol-9 gel against STI infection, conducted among Cameroonian sex workers, found decreased reports of condom use one year following trial exit [1].

Here, we present data on barrier method use several months after the Methods for Improving Reproductive Health in Africa (MIRA) trial among Zimbabwean participants recruited from the general population. The MIRA trial evaluated the effectiveness of the diaphragm against HIV/STI acquisition. Diaphragms are commercially available worldwide as one of the oldest contraceptive methods [2]. As previously described [3], during the MIRA trial, all participants received a comprehensive HIV prevention package consisting of: pre-test and post-test counselling about HIV and STIs; testing and treatment of curable STIs; and intensive risk-reduction counselling that included education, demonstration and promotion of male condom use during every sex act, provision of free male condoms, and provision of a fact sheet with instructions on how to use condoms.

All volunteers were also fitted with diaphragms, received instructions and practiced insertion of the diaphragms at the clinic to ensure that women in the diaphragm group were not inherently better at using the diaphragm than those in the condom group. After randomization, women in the diaphragm group received education and counselling about the diaphragm and a product instruction sheet. HIV/STI testing and counselling and risk-reduction counselling were repeated at every follow-up visit, as was product counselling (condoms or condoms and diaphragm) as appropriate for group assignment.

Main results from the trial have been previously published, and no significant protective effect of the intervention against HIV or STI could be demonstrated [3-5]. We also previously examined diaphragm adherence in the MIRA intervention sample [6]. For this analysis, we focused on post-trial use of male condoms, diaphragms and any barrier methods (condoms and/or diaphragm) among Zimbabwean MIRA participants. Specifically, we assessed post-trial use compared with use during the trial. Additionally, we compared post-trial use with that reported at trial enrolment.

## Methods

This analysis draws data from the MIRA trial, an open-labelled, multisite, randomized, controlled trial of the diaphragm and Replens^® ^lubricant gel in South Africa and Zimbabwe. It also draws data from an ancillary study, which consisted of a cross-sectional post-trial study visit among a subset of former MIRA participants at the Zimbabwean site to validate reports of recent sexual activity and method use using a biomarker of semen exposure (prostate-specific antigen). Detailed methods for recruitment, eligibility criteria, study procedures and main findings for both these studies have been published elsewhere [3,7].

### Study setting and population

The MIRA trial was conducted between 2003 and 2006 (registration with http://ClinicalTrials.gov, number NCT00121459). Women were recruited from reproductive and general health clinics and the community. Eligibility criteria included being: 18-49 years old; HIV uninfected; non-pregnant; sexually active; free of treatable STIs, with a healthy cervix; and able to insert the diaphragm prior to randomization. Participants were seen at two study clinics within 30 kilometres of Harare (Chitungwiza, a peri-urban municipality, and Epworth, a slightly poorer and less developed suburb), and followed between 12 and 24 months (depending on their calendar date of enrolment). The retention rate for MIRA Zimbabwean participants was 94.2%.

#### Study procedures

At MIRA screening, following written informed consent, all volunteers provided demographic information, received HIV/STI testing and counselling, received treatment of curable STIs, and were provided with free male condoms [3]. Approximately two weeks after screening, eligible women returned for their enrolment visit and were randomized into a diaphragm group (receiving diaphragm, gel and male condoms) or a condom group (receiving male condoms only). At enrolment and quarterly thereafter, participants received: behavioural assessments in their native language using Audio Computer-Assisted Self-Interviewing (ACASI); HIV testing with pre- and post-test counselling; risk-reduction counselling that included use of male condoms during every sex act; and free male condoms.

##### Diaphragm education and provision

All women were fitted by a trained study clinician, received a diaphragm educational session, and successfully practiced diaphragm insertion and removal at the clinic prior to randomization. Additionally, women in the diaphragm group received quarterly diaphragm adherence counselling, as previously described [6]. So as not to discourage participation among women randomized to the control group, and because the study products were commercially available, all participants were told they could obtain diaphragms and gel after study completion.

##### Trial exit procedures

All participants received free male condoms at their MIRA exit visit, and were encouraged to return to the clinic for resupply of condoms. At MIRA study exit, women in the diaphragm group could elect to keep their study diaphragms or be refitted and receive new devices (if they had been fitted a year or more previously) and receive a year's supply of study gel (commensurate with their coital frequency). Similarly, women in the condom group could elect to take study diaphragms, and each of those interested was fitted, received a diaphragm and a supply of study gel. All exiting participants who elected to keep or receive diaphragms received a comprehensive educational session, emphasizing that trial results were not yet known and reviewing what was known and unknown about the diaphragm. Before supplies were dispensed, participants completed a comprehension quiz and had to demonstrate full understanding that it was not a proven method for HIV/STI prevention or contraception (when used with a non-spermicidal gel) [8].

Post-trial product distribution procedures were discussed extensively by the study's scientific team and approved by all institutional review boards, community advisory boards and ethical consultants to the study. In late July and August 2007, when participants were informed of the final MIRA trial results, which showed no effect of the intervention, participants were discouraged to continue use of the diaphragm. Staff attempted to contact all participants and invited them to come to the study results meetings at which investigators explained the results and answered questions. The post-study visit described in the next section took place prior to the release of trial findings.

#### Post-trial visit

We conducted a cross-sectional ancillary study to validate self-reports of recent sexual activity using a biomarker, which included one post-trial visit conducted between December 2006 and June 2007, and enrolled a subset of MIRA participants from Zimbabwe. Only non-pregnant, former MIRA participants without a vaginal delivery or third-trimester stillbirth in the prior six weeks were eligible for enrolment. Women learned about this ancillary study at their last MIRA visit, through community outreach or during drop-in clinic visits that occurred after completing the trial (e.g., to obtain additional condoms). For the ancillary study, women were randomized in approximately equal numbers into one of two interview modalities: ACASI (n = 450) or face-to-face interview (FTFI) (n = 460). Since the baseline characteristics of women in the ACASI and FTFI groups were similar and results from the two modalities were not statistically different [7], we conducted combined analysis of *all *behavioural responses from the ancillary study. Nonetheless, we adjusted for interview mode at the post-trial visit in all multivariable analyses to control for possible unmeasured confounding.

#### Study sample

**Figure 1:**
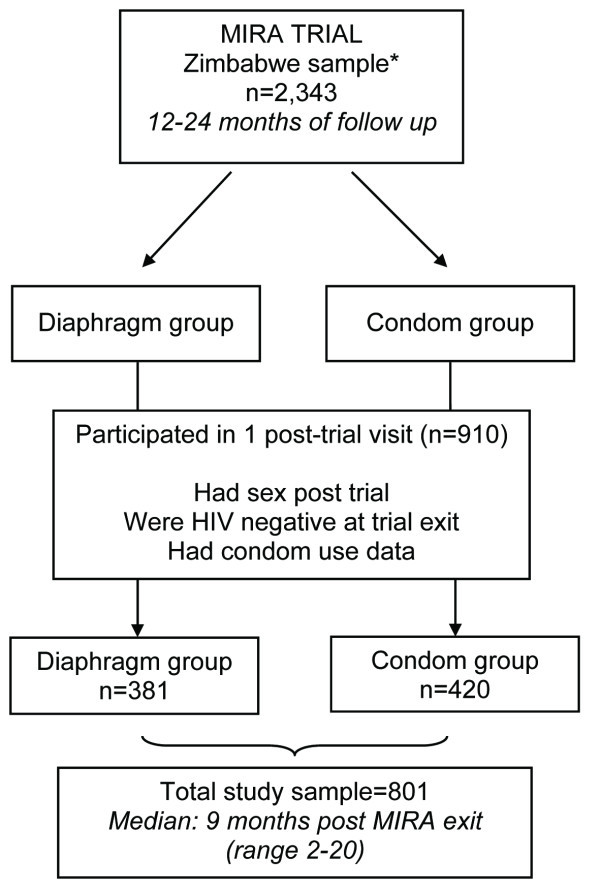
**Study sample flow chart**. *This includes women who remained HIV seronegative throughout the duration of the trial, and excludes those (n = 31) who had no MIRA follow-up data.

The original ancillary study included 910 former MIRA Zimbabwean participants [7]. Of those, 840 had sex since completing the MIRA study (92.3%); 803 had not HIV seroconverted during the MIRA trial; and 801 women, our final analysis sample, had condom use data at one or more MIRA follow-up visits (see Figure 1).

#### Measures

Barrier method(s) use at last sex act was assessed at enrolment, at every MIRA quarterly visit (using ACASI) and at the post-trial visit (using ACASI or FTFI). For this study, our *main outcome measures *were: (a) male condom use at last sex act (yes/no); (b) protected last sex act by a barrier method (male condom, female condom or diaphragm) (yes/no); and (c) for MIRA diaphragm group participants only, diaphragm use at last sex (yes/no).

##### Exposure measures

We created binary indicator variables for each study visit type. For our primary analysis, we compared method use at the last MIRA follow-up visit versus the post-trial visit. Second, we compared method use at MIRA enrolment versus post trial.

##### Covariates

Because we hypothesized that the time lapse between the final MIRA follow-up visit and the post-trial visit could affect reported method use, we created a continuous time since exit indicator variable by calculating the time (in months) between a participant's last MIRA study visit and her post-trial visit (range: two to 20 months). To assess a dose effect from repeated counselling at regular MIRA visits, we also created a continuous variable of the number of completed quarterly follow-up visits in MIRA that a participant had received (range: one to eight). In all multivariable analyses, we controlled for post-trial visit interview mode (ACASI vs. FTFI) and age (as a continuous measure). We also examined the following additional potential confounders: baseline education, marital status, cohabitation with main partner, lifetime partners, and having any new partner during the MIRA trial. We found no evidence that these additional covariates confounded the main associations of interest and thus did not include them in the multivariable analyses. To address possible confounding from acquiring an STI during the trial, we conducted a sensitivity analysis where participants who were diagnosed with chlamydia, gonorrhea or trichomonas during MIRA were removed from the sample. The results were essentially identical (data not shown).

#### Analyses

Preliminary analyses were descriptive and focused on the proportion of participants at a given visit type who used a specific barrier method (male condom; diaphragm; female condom) or any barrier method at last sex. At baseline, we examined socio-demographic differences and behavioural characteristics between our study sample and the remaining Zimbabwe MIRA sample, using chi-square tests for categorical variables, t-tests or Wilcoxon rank-sum tests for continuous variables, and Poisson regression for count variables.

To examine changes between visits in method(s) used at last sex, we compared study outcomes for each participant at the last MIRA follow-up visit and the post-trial visit, using multivariable random effects logistic regression models. As we anticipated differing patterns in each MIRA study group, separate models were run for the diaphragm and the condom groups. Using a similar approach, we also examined change in method(s) used at last sex between MIRA enrolment and the post-trial visit. All analyses were conducted using SAS version 9.1 (SAS Institute, Inc., Cary, NC, USA).

#### Ethical approval

All participants provided written informed consent prior to participating in MIRA and the ancillary study. The following local ethics committees at collaborating institutions gave approval for the studies: the institutional review boards at the University of California, San Francisco; the Medical Research Council of Zimbabwe; the Medicines Control Authority of Zimbabwe; the Western IRB; and Family Health International. The MIRA study is registered with http://ClinicalTrials.gov (number NCT00121459).

## Results

### Study sample

This analysis includes data from 801 former MIRA trial Zimbabwean participants. As shown in Table 1, the majority of the women were 25 years old and above, and more than half had not completed high school. Women had a median of one lifetime partner (range: one to five) and almost all were married and cohabitating with their partners. More than three-quarters used hormonal contraceptives at baseline, and the distribution of contraceptive method use was similar at trial exit. "Ever use" of male condoms increased from 65.5% at screening to 91.0% at enrolment (McNemar test, p < 0.0001). At enrolment, 74% reported using a barrier method at their last sex act, with 71.0% using a male condom and 3.4% a female condom. Women completed a median of eight quarterly follow-up visits during MIRA (range: one to eight), and there was a median of nine months (range: two to 20) between women's final MIRA visits and their post-trial visits.

**Table 1:** Characteristics of study sample at MIRA baseline, exit visit and post-trial visit; n = 801

		Study sample	
		n = 801	%
**Baseline**			

Randomization arm	Intervention	381	47.6
	Control	420	52.4
			
Age group	18-24	261	32.6
	25-34	379	47.3
	35+	161	20.1
Education (binary)	< High school	447	55.8
Married	Yes	775	96.8
Cohabitating	Yes	777	97.0
Lifetime partners (1 vs. > 1)	One lifetime partner	628	78.4
Ever had vaginal sex using a male condom (screening)	Yes	525	65.5
Ever had vaginal sex using a male condom (enrolment)	Yes	728	91.0
Condom use in the past 3 months (enrolment)	Always	215	26.8
	Sometimes	356	44.4
	Never	230	28.7
Male condom use at last sex (enrolment)	Yes	569	71.0
Ever female condom use (enrolment)	Yes	58	7.2
Female condom use at last sex (enrolment)	Yes	27	3.4
Last sex act protected by a barrier method (enrolment)	Yes	590	73.7
Ever used diaphragm	Yes	1	0.1
Coital frequency per week at screening (≤3, > 3)	≤3	423	52.8
Main contraceptive at screening (#)	Long term	25	3.1
	Injectable	113	14.1
	Pill	514	64.2
	Barrier method	95	11.9
	Other/none	54	6.7

**MIRA follow up and exit**			

Number of quarterly follow-up visits in MIRA	Mean; median (range)	6.83; 8 (1-8)	
Ever had a new regular partner during MIRA trial	Yes	212	26.5
Kept/took diaphragm at exit visit (entire sample)	Yes	579	72.3
Kept/took diaphragm in intervention arm (at exit visit) n = 381	Yes	369	96.9
Took diaphragm in control arm (at exit visit) n = 420	Yes	210	50.0
Main contraceptive at exit visit (#)	Long term	26	3.3
	Injectable	139	17.4
	Pill	457	57.1
	Barrier method	108	13.5
	Other/none	71	8.9

**Post-trial study visit**			

Months between last MIRA visit and post-trial visit	Mean; median (range)	9.41; 9 (2-20)	
	**Total n**	**n**	%
Male condom use at last sex	801	497	62.1
Female condom use at last sex	801	21	2.4
Diaphragm use at last sex (intervention arm)	381	192	50.4
Diaphragm use at last sex (intervention arm)*	369	190	51.5
Diaphragm use at last sex (control arm)*	210	31	14.8
Diaphragm and male condom use at last sex (intervention arm)	381	123	32.3
Diaphragm and male condom use at last sex (intervention arm)*	369	122	33.1
Diaphragm and male condom use at last sex (control arm)*	210	10	4.8
Protected last sex act	801	597	74.5

*among those who took/kept the diaphragm at MIRA exit visit

(#) hierarchical categorization

This study sample was very similar to MIRA Zimbabwean participants who did not participate in this study. However, women in this study were slightly older (32.6% were 18-24 vs. 38.1%; p = 0.02), had lower educational attainment (55.8% did not complete high school vs. 49.1%; p = 0.02), were more consistent product users (condoms: OR = 1.34, 95% CI: 1.18-1.53, and diaphragm: OR = 1.30, 95% CI: 1.09-1.54) and attended more MIRA follow-up visits (median eight vs. six visits, p < 0.0001) compared with MIRA Zimbabwean participants who did not join this study (data not shown).

### Patterns of method(s) use at last sex

#### Male condoms

**Figure 2:**
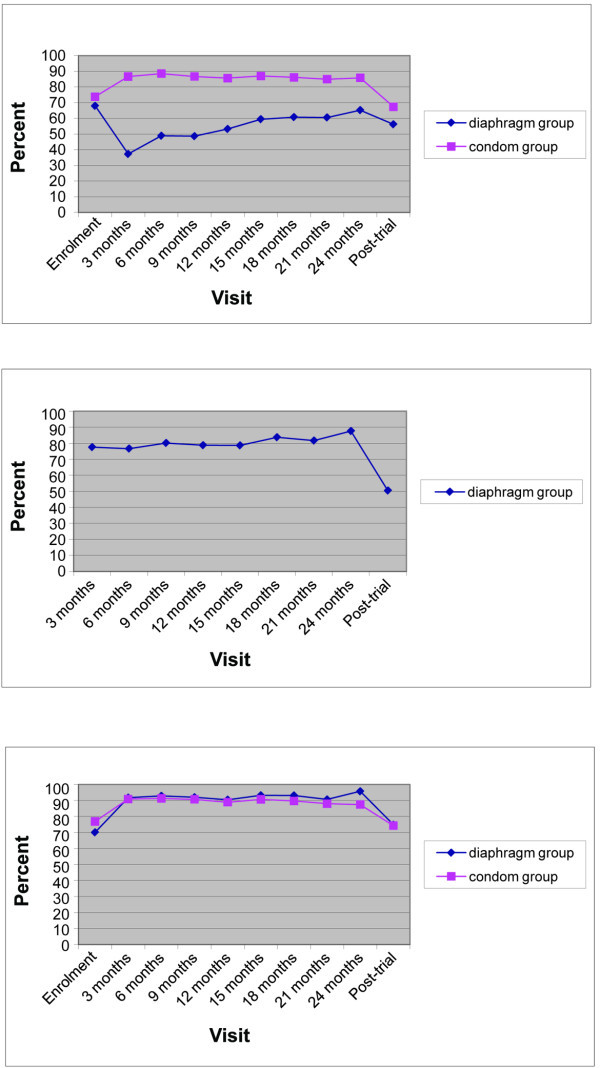
**Group averages in method(s) used at last sex, by MIRA visits and post-trial visit**. Figure 2a. Male condom use at last sex by visit type and MIRA diaphragm and condom groups; n = 801 Figure 2b. Diaphragm use at last sex by visit type in MIRA diaphragm group; n = 381 Figure 2c. Barrier method(s) use at last sex act (male condom, female condom, diaphragm) by visit type and MIRA diaphragm and condom groups; n = 801

As shown in Figure 2a, group averages among women in the MIRA condom group indicate that male condom use at last sex increased between enrolment (73.8%) and the first quarterly visit (86.7%), and decreased between the MIRA 24-month visit (85.8%) and the post-trial visit (67.4%). As reported for the multisite MIRA sample [3], in the diaphragm group, condom use at last sex showed a different temporal trend between MIRA enrolment (68.0%), first quarterly visit (37.3%) and the 24-month visit (65.2%); condom use also decreased at the post-trial visit (56.2%).

#### Diaphragm

Only one woman reported ever using a diaphragm prior to study entry (Table 1). At trial exit, almost all women in the diaphragm group (n = 369; 96.9%) kept their diaphragms or elected to be fitted with new ones. As shown in Figure 2b, in the diaphragm group, there was a slight increase in diaphragm use at last sex during the trial: from 77.7% at the first quarterly visit to 88.6% at the 24-month visit, and then decreasing to 50.4% at the post-trial visit. In the condom arm, 210 women (50%) elected to be fitted for diaphragms at trial exit. Among these, 31 (14.8%) reported using diaphragms at last sex at their post-trial visit (Table 1).

#### Barrier method use (male condom, female condom and/or diaphragm) at last sex act

**Figure 3:**
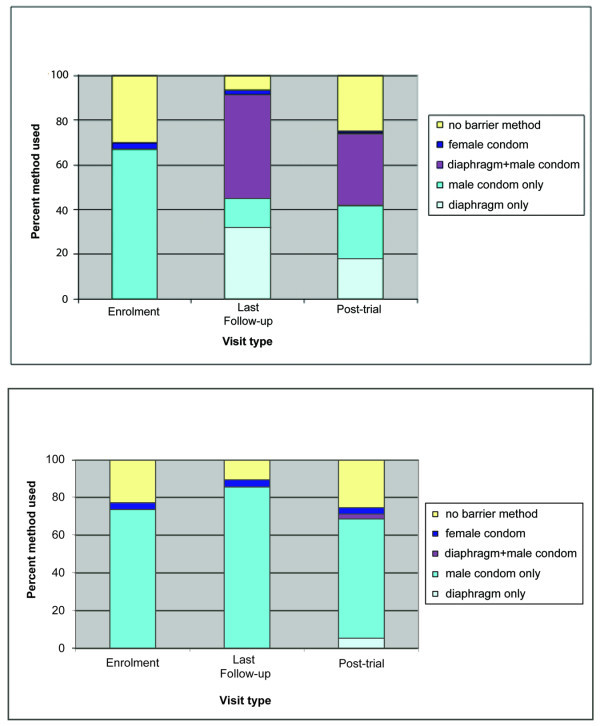
**Last sex act by barrier method used and by visit type**. Figure 3a. Last sex act by barrier method used and by visit type among diaphragm group participants; n = 381 Figure 3b. Last sex act by barrier method used and by visit type among condom group participants; n = 420

As shown in Figure 2c, patterns of barrier method use at last sex act were very similar between the diaphragm and condom groups, showing the most increase between enrolment and the first quarterly follow up. At the MIRA 24-month visit, 95.7% in the diaphragm group and 87.4% in the condom group reported using a barrier method at their last sex act. This decreased to 74.8% and 74.3% at the post-trial visit, respectively. The mix of barrier methods used at last sex for different visit types (MIRA enrolment, final trial visit and post-trial visit) is summarized in Figures 3a and b.

In the condom group, as expected, male condoms contributed most to the barrier methods mix across all visit types. In contrast, in the diaphragm group, at final MIRA visit, 32% of last sex acts were protected by the diaphragm only; this decreased to 18.1% at the post-trial visit. Diaphragm and condom used together (per MIRA protocol requirement) were reported by 46.5% participants at their last MIRA visits and by 32.3% at the post-trial visit. In the diaphragm group, male condom used alone was reported by 66.9% of women at enrolment, 13.1% at final trial visit and 23.6% at the post-trial visit.

### Multivariable analysis of individual-level changes in reported method use

#### Condom group

As shown in Table 2, between a participant's last MIRA visit and her post-trial visit, there was a significantly decreased odds in her report of condom use at last sex (AOR = 0.20; 95% CI 0.12-0.33; p < 0.0001) and of use of any barrier method at last sex (AOR = 0.21; 95% CI 0.12-0.33; p < 0.0001). These findings were not affected by the number of months between trial exit and the post-trial visit, nor by the number of MIRA visits attended.

**Table 2:** Change in barrier method(s) use at last sex, by visit type and by study groups

Change in condom use and use of any barrier method at last sex, among condom group participants (n = 420)									
**Last MIRA Follow-up Visit**		**Last FU visit**		**Post-trial visit**		**AOR**	**Lower limit**	**Upper limit**	**p value**
		**n**	%	**n**	%				
model 1	**Condom use at last sex**	363	86.43	283	67.38				
	Visit type*					0.20	0.12	0.33	< 0.0001
	Time since trial exit					0.95	0.88	1.03	0.228
	Number of MIRA visits					1.01	0.84	1.22	0.8899
model 2	**Any barrier method**	374	89.05	312	74.29				
	Visit type*					0.21	0.12	0.35	< 0.0001
	Time since trial exit					0.96	0.88	1.05	0.347
	Number of MIRA visits					0.93	0.75	1.16	0.5293
**MIRA Enrolment Visit**		**Enrolment visit**		**Post-trial visit**		**AOR**	**Lower limit**	**Upper limit**	**p value**
		**n**	%	**n**	%				
model 1	**Condom use at last sex**	310	73.81	283	67.38				
	Visit type**					0.66	0.46	0.93	0.0196
	Time since trial exit					0.96	0.90	1.02	0.1824
	Number of MIRA visits					1.07	0.91	1.25	0.4349
model 2	**Any barrier method**	323	76.9	312	74.29				
	Visit type**					0.83	0.58	1.19	0.3178
	Time since trial exit					0.96	0.90	1.02	0.2077
	Number of MIRA visits					1.02	0.87	1.20	0.7732
**Change in diaphragm, condom use and use of any barrier method at last sex, among diaphragm group participants (n = 381)**									

**Last MIRA Follow-up Visit**		**Last FU visit**		**Post-trial visit**		**AOR**	**Lower limit**	**Upper limit**	**p value**
		**n**	%	**n**	%				
model 1	**Diaphragm at last sex**	303	79.53	192	50.39				
	Visit type*					0.18	0.12	0.28	< 0.0001
	Time since trial exit					0.95	0.89	1.01	0.1058
	Number of MIRA visits					1.28	1.09	1.49	0.0024
model 2	**Condom use at last sex**	231	60.63	214	56.17				
	Visit type*					0.77	0.55	1.09	0.1425
	Time since trial exit					1.03	0.96	1.10	0.4054
	Number of MIRA visits					1.26	1.07	1.49	0.0062
model 3	**Any barrier method**	357	93.7	285	74.8				
	Visit type*					0.15	0.08	0.27	< 0.0001
	Time since trial exit					0.93	0.86	1.00	0.0593
	Number of MIRA visits					1.29	1.07	1.55	0.0072
**MIRA Enrolment Visit**		**Enrolment visit**		**Post-trial visit**		**AOR**	**Lower limit**	**Upper limit**	**p value**
		**n**	%	**n**	%				
model 1	**Condom use at last sex**	259	67.98	214	56.17				
	Visit type**					0.55	0.39	0.76	0.0004
	Time since trial exit					0.99	0.94	1.05	0.7865
	Number of MIRA visits					1.18	1.03	1.36	0.0153
model 2	**Any barrier method**	267	70.08	285	74.8				
	Visit type**					1.33	0.93	1.89	0.1134
	Time since trial exit					0.96	0.90	1.02	0.1764
	Number of MIRA visits					1.16	1.00	1.34	0.0553

AOR = adjusted odds ratio; CL = confidence limit; FU = follow up

*post-trial visit vs. last MIRA follow-up visit

**post trial visit vs. MIRA enrolment visit

All models are controlling for age and substudy interview mode group (ACASI vs. FTF I)

#### Diaphragm group

As shown in Table 2, between a participant's last MIRA visit and her post-trial visit, there was a significantly decreased odds in her report of diaphragm use at last sex act (AOR = 0.18; 95% CI 0.12-0.28; p < 0.0001) and of use of any barrier method at last sex (AOR = 0.15; 95% CI 0.08-0.27, p < 0.0001). Reported condom use at last sex decreased non-significantly between these two visit types. In the diaphragm group, the more MIRA visits a woman attended, the more likely she was to report use at last sex for each of the three outcomes: male condoms, diaphragm, and any barrier method. The number of months between trial exit and the post-trial visit did not influence these outcomes.

When assessing method use at enrolment compared with the post-trial visit, similar results were found for the condom and diaphragm groups (Table 2): there was a significantly decreased odds in a woman's report of condom use at last sex act. However, there was no difference in a woman's odds of reporting any barrier method use at enrolment and at her post-trial exit visit. In the diaphragm group only, the number of MIRA visits was associated with these outcomes, but not the number of months between trial exit and the post-trial visit.

## Discussion

This study is among the few that report patterns of male condom and other barrier method use by women after participating in an HIV prevention trial. We compared self-reported condom use at enrolment (but before randomization), at the end of the trial, and several months after trial completion. Because the diaphragm (our investigational product) is commercially available, participants who elected to do so were allowed, after careful and comprehensive education and counselling, to take diaphragms at MIRA trial exit. Thus, we also had unique data on intervention and post-intervention use of the diaphragm.

In the condom group, reported condom use at last sex significantly decreased between the MIRA last study visit and the post-trial visit. Furthermore, there was a small but significant decrease between condom use at MIRA enrolment compared with the post-trial visit. Also, more MIRA visits did not influence reported condom use, nor did the time elapsed between MIRA exit and the post-trial visit. Taken together, these results suggest that there was no sustained effect of repeated counselling on condom use after the counselling and study participation stopped. At MIRA trial exit, women received condoms and were encouraged to return to the clinic for additional free condoms. They also were provided with referrals for obtaining condoms free of charge. Still, passive access may not have been sufficient to maintain high levels of condom use post trial, and women or partners' willingness to use condoms after the trial may have decreased. During the trial period, sustained behaviour change may have been maintained by regular HIV testing, ongoing risk-reduction counselling, perceived obligation among participants and their partners to use condoms while in the study, as well as free condom provision.

Diaphragms were evaluated in MIRA for disease prevention, and were mostly unavailable and virtually unused as contraceptives in the geographical areas where the trial was conducted [9]. This gave us a unique opportunity to assess uptake and post-trial use of a previously unknown female-initiated method. Based on the principles of participants' autonomy and right to choose, and given that the device is safe and commercially available, each woman was allowed to take a diaphragm at trial exit. Unfortunately, when trial results became available, findings failed to demonstrate significant protection to users, and participants were advised to stop using the device.

Only half of the condom group elected to take diaphragms at MIRA trial exit, and of those, a small minority reported using them at their post-trial visit. This is not surprising as diaphragms were provided at MIRA exit with a great deal of cautionary information, emphasizing their unknown effectiveness against HIV/STIs or pregnancy (when used without a contraceptive gel). Interestingly, most women in the diaphragm group, who received the same exit information and education, chose to keep and/or take the diaphragms at trial exit, and half still reported diaphragm use (at last sex) several months after the trial. We interpret this as an indication of genuine acceptability of the device among users, and this is corroborated by high reported acceptability during the trial [10] and in other studies of cervical barriers [11,12]. Alternatively, our exit product education and counselling may have been less effective than intended.

The difference in diaphragm use at the post-trial visit between the condom and diaphragm groups, both of which had the option of receiving diaphragms at their exit visit, highlights that access and basic skills taught at the clinic may not be sufficient for uptake of a new method and that "real-life" experience, along with ongoing support, may better ensure uptake and continued use. Previous reports from other developing countries indicate that adequate information and support, as well as good user skills, are required to ensure diaphragm uptake and to avoid high discontinuation rate as a contraceptive [13,14]. Additionally, in a previous Zimbabwean study assessing the diaphragm as a potential disease prevention method, problems with the device significantly decreased over time, suggesting that practice with the device and educational follow-up will help improve user skills [11].

Reported use of any barrier method at last sex was highest (almost 75%) at the post-trial visit, and compared with MIRA trial exit visit levels, it decreased less than condom or diaphragm use individually. Furthermore, levels were similar to those reported at MIRA enrolment for women in both the condom group and the diaphragm group. As has been shown in other studies, expanding the mix of methods increases method coverage [15,17].

We previously reported that protocol-required concurrent use of two barrier methods (male condoms and diaphragms) proved challenging during the trial, as highlighted by a drop in reported male condom use in the diaphragm group during MIRA follow up [18,19]. This drop was most pronounced at the first quarterly follow-up visit; then there was a gradual increase in condom use over the MIRA follow-up period, although reported condom use in the diaphragm group never reached enrolment levels. Our data suggest that in the diaphragm group, more exposure to testing, counselling and educational messages was associated with an increased likelihood of reporting use of male condoms, diaphragms or any barrier method. Since women in the diaphragm group were introduced to a "new" method, repeated education and adherence counselling may have translated into progressive skill acquisition and increased use. Ongoing support and counselling may have also somewhat encouraged condom use after the initial drop, possibly by helping women to overcome some of the challenges they faced with concurrent use of diaphragms and condoms [19].

There are several limitations to this study. Most importantly, method(s) use was self-reported and may have been influenced by recall and social desirability biases. We only examined product use "at last sex" because others have reported that it is representative of behaviour over longer time periods, such as "use in the last three months" (Ben Masse, personal communication, 2008 and [20]), but minimizes recall bias.

Second, we cannot tease out if the increase in condom use reported during MIRA follow up in the condom group was due to the effect of condom counselling, a trial effect, or to over-reporting of a socially desirable behaviour. Although results from the ancillary study indicate that self-reported condom use was likely inflated [7], we didn't expect this bias to be differential between visit types, and thus, individual-level analyses of relative changes over time should still provide useful insights into participants' behaviour. The same holds true for diaphragm reports in the diaphragm group. Indeed, because MIRA enrolment occurred after one or more screening visits involving intensive interaction with clinical staff, and the post-trial visit was conducted at the MIRA study clinic with the same clinical staff, we had no reason to expect that if misreporting occurred, it would significantly differ by visit type.

Third, we started assessing our behavioural measure of method use at last sex at the enrolment visit, after women had already been screened, HIV tested and counselled about condoms. Thus, the baseline condom use reported here was likely higher than in a study-naïve population, and indeed, we did observe that "ever use of condoms" significantly increased between MIRA screening and enrolment visits [3]. Thus, post-trial use of condoms, though lower than use during the trial might have remained higher than condom use prior to study entry. It is not clear what would help sustain male condom use post trial. Our results suggest that some level of services, including counselling, should be continued beyond the duration of the trial.

Somewhat unexpectedly, diaphragm use persisted after women exited the trial, especially among women in the diaphragm group who were experienced users. Thus, availability of trained staff for support, user skills and habituation may be important for sustaining "new" product use. In contrast to condoms, access to a diaphragm did not present a barrier to use since it is reusable, and this may have facilitated continued use after the trial.

## Conclusions

High condom use levels achieved during the trial were not sustained post trial in the condom group. Post-trial diaphragm use remained relatively high in the diaphragm group (given its unknown effectiveness), but was very low in the condom group. Introducing "new" methods for HIV prevention may require time and user skills before they get adopted. Our findings underscore the potential benefit of providing a mix of methods to women as it may encourage more protected acts. We anticipate that the findings presented here may inform better design of behavioural research in the context of biomedical clinical trials, which will generate more reliable and useful outcomes to guide operations research and programmatic rollouts.

## Competing interests

The authors declare that they have no competing interests.

## Authors' contributions

AVDS conceived of the study, participated in the development of the study protocols, led the analysis, and drafted the manuscript. HC performed the statistical analysis. AMM participated in the development and design of the project and protocol, provided scientific input in analysis, and reviewed and edited the manuscript. All co-authors read and approved the final version of the manuscript.
